# CHOP Regulates Endoplasmic Reticulum Stress-Mediated Hepatoxicity Induced by Monocrotaline

**DOI:** 10.3389/fphar.2021.685895

**Published:** 2021-05-24

**Authors:** Yazhou Guo, Chen Yang, Rong Guo, Ruijie Huang, Yongxia Su, Shuai Wang, Yezi Kong, Jianguo Wang, Chengjian Tan, Chonghui Mo, Chenchen Wu, Baoyu Zhao

**Affiliations:** ^1^ College of Veterinary Medicine, Northwest A&F University, Yangling, China; ^2^ Institute of Poisonous Plants in Western China, Northwest A&F University, Yangling, China; ^3^ Department of Biotechnology, Guizhou Minzu University, Guiyang, China; ^4^ College of Agriculture and Animal Husbandry, Qinghai University, Xining, China

**Keywords:** monocrotaline, hepatotoxicity, endoplasmic reticulum stress, CHOP, apoptosis

## Abstract

Monocrotaline (MCT), a pyrrolizidine alkaloid, is the major toxin in *Crotalaria*, which causes cell apoptosis in humans and animals. It has been reported that the liver is a vulnerable target of MCT. However, the exact molecular mechanism of the interaction between endoplasmic reticulum (ER) stress and liver injury induced by MCT is still unclear. In this study, the cytotoxicity of MCT on primary rat hepatocytes was analyzed by a CCK-8 assay and Annexin V-FITC/PI assay. Protein expression was detected by western blotting and immunofluorescence staining. As a result, MCT significantly decreased the cell viability and mediated the apoptosis of primary rat hepatocytes. Meanwhile, MCT could also induce ER stress in hepatocytes, indicated by the expression of ER stress-related proteins, including GRP78, p-IRE1α, ATF6, p-eIF2α, ATF4, and CHOP. Pretreatment with 4-PBA, an inhibitor of ER stress, or knockdown of CHOP by siRNA could partly enhance cell viability and relieve the apoptosis. Our findings indicate that ER stress is involved in the hepatotoxicity induced by MCT, and CHOP plays an important role in this process.

## Introduction

Pyrrolizidine alkaloids (PAs) are a common group of chemical constituents, and more than 660 PAs and PA N-oxides have been identified. As reported, PAs are distributed in over 6,000 plants from 13 distantly related angiosperm families, and Fabaceae, Compositae, and Boraginaceae are the dominant species (Xia et al., 2016; Ahmad et al., 2018; Zhu et al., 2018). To date, hepatotoxicity is a hallmark of PAs and about half of them are poisonous compounds affecting livestock, wildlife, and humans (Wiedenfeld, 2011; Xia et al., 2016). In fact, to survive, plants use PAs as a chemical defense to against herbivores (Castells et al., 2017). Therefore, livestock and wildlife over intake of PA-containing plants is hazardous for their health. Meanwhile, humans could be exposed to PAs through herbal medicine and contaminated food, such as milk, honey, and tea. So, PAs are already a threat to public health (Edgar et al., 2015).

Monocrotaline (MCT), a representative PA toxin, is biosynthesized in plants of the genus *Crotalaria*, belonging to Fabaceae (Yang et al., 2017a). Numerous MCT poisoning cases have been reported in livestock and humans (Lyford et al., 1976; Guan, 2006; Pessoa et al., 2013; Robinson and Gummow, 2015). The damages from MCT exhibit acute toxicity and chronic toxicity. In acute cases, the liver shows hemorrhagic necrosis, veno-occlusion, and hepatic carcinomas (Edgar et al., 2015). In chronic exposure, MCT might damage multiple organs, such as the liver, lungs, kidneys, and brain (Stegelmeier et al., 2016; Yang et al., 2017a). However, as a vital target of MCT stimulation, the potential mechanism of action for MCT-induced hepatocyte lesions has not been completely clarified.

Presently, the important role of the endoplasmic reticulum (ER) to respond to perturbations of xenobiotics is critical for cell survival (Iurlaro and Muñoz-Pinedo, 2016). When the homeostasis balance in the ER is disturbed by various factors, including alterations in calcium stores, hypoxic conditions, and disturbances to the redox balance, ER stress is triggered. In the early stage, cells adapt to this stress by stopping protein synthesis and promoting ER-associated degradation (ERAD) to recover the internal environment to homeostasis. In contrast, if the stimulation exceeds the control of cell ability, ER stress-triggered cell death could be induced (Tabas and Ron, 2011). In eukaryotic cells, protein kinase RNA-like ER kinase (PERK), inositol requiring enzyme-1α (IRE1α), and activating transcription factor-6α (ATF6α) were divided from the molecular chaperone GRP78 in ER stress, permitting transduction of downstream signals and initiating the expression of CHOP. The overexpression of CHOP plays a critical role in the process of apoptosis (Rao et al., 2015; Huang et al., 2021). Meanwhile, more and more researchers are focusing on the diseases caused by ER stress and suggest that ER stress could mediate other factors to induce liver injury (Iracheta-Vellve et al., 2016; Han et al., 2018; Torres et al., 2019). Therefore, we propose the hypothesis that ER stress can be considered an underlying mechanism when evaluating the toxicity of MCT.

While our previous study suggested that MCT could induce ER stress in rat livers (Guo et al., 2020), it is not known whether ER stress participates in MCT-induced hepatocyte apoptosis as well. In this study, we confirmed that ER stress was involved in MCT-induced hepatotoxicity. Meanwhile, we further demonstrated that CHOP was a vital factor for apoptosis when treating MCT.

## Materials and Methods

### Reagent

**FIGURE 1:**
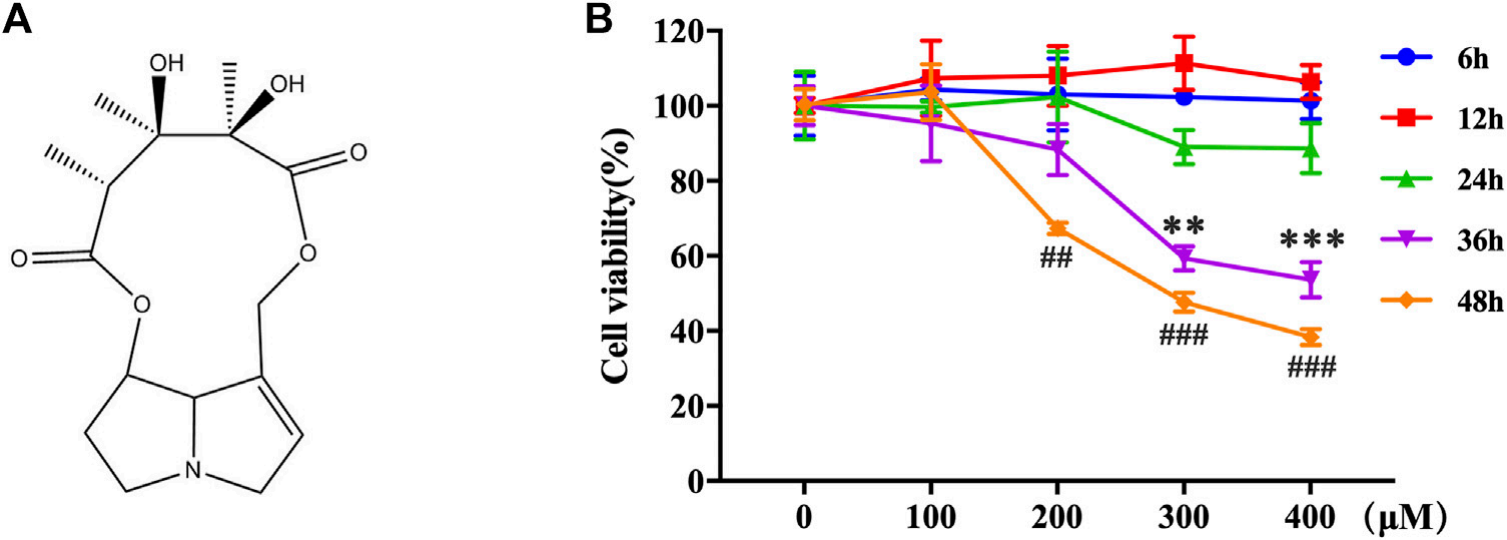
MCT decreased the cell viability in primary rat hepatocytes. **(A)** The chemical structure of MCT. **(B)** Cell viability was measured by CCK-8 assay. The hepatocytes were treated with different doses of MCT (100, 200, 300, and 400 μM) for different times (6, 12, 24, 36, and 48 h). The results are represented in the way of mean ± SD error of three independent experiments. **p < 0.01 and ***p < 0.001 compared to the control of the 300 μM group, ^##^ *p* < 0.01 and ^###^ *p* < 0.001 compared to the control of the 400 μM group.

Monocrotaline (MCT, Figure 1A, purity > 98%, CAS No.: 315-22-0) was purchased from Sigma Aldrich (United States, Catalog No.: C2401) and a stock concentration of 50 mM of MCT was prepared by dissolving in 1 mol/L of HCl and balancing the pH to 7.0–7.4 by adding 5 mmol of NaOH. DMEM medium (Gibco, United States, Catalog No.:12800017) containing 10% FBS (Zeta Life, United States, Catalog No.: Z7181FBS) was used for cell culture. 4-phenylbutyric acid (4-PBA) was purchased from MedChemExpress Co., Ltd (China, Catalog No.: HY-15654).

### Isolation, Culture, and Treatment of Rat Primary Hepatocytes

Rat primary hepatocytes were isolated using the standard two-step perfusing procedure according to Slegen (Seglen, 1976). Briefly, a male Sprague–Dawley rat was obtained from Cheng Du Dossy Biological Technology Co., Ltd. (Sichuan, China) and was anesthetized with pentobarbital (100 mg/kg; i.p.), and the liver was perfused through a needle aligned along the portal vein, with perfusion solution A (140 mM NaCl, 6.7 mM KCl, 2.5 mM glucose, 10 mM HEPES, and 0.5 mM EGTA); followed by perfusion solution B (140 mM NaCl, 6.7 mM KCl, 2.5 mM glucose, 30 mM HEPES, 5 mM CaCl_2_), containing 0.5 mg/ml of collagenase IV (Gibco, CAS No.: 9001-12-1, Catalog No.: 17104-019). Then, the perfused liver was separated and suspended in DMEM media without FBS. The suspended hepatocytes were filtered through a 75-μm nylon membrane and centrifuged (23 × *g*, 5 min at 4°C) twice. Afterward the hepatocytes were purified using density gradient centrifugation (50% Percoll solution, 211 × *g* for 10 min at 4°C). Then, the hepatocytes were resuspended in DMEM with 10% FBS. Isolated hepatocytes were seeded at a density of 1 × 10^5^ cells/100 μL in 96-well plates for toxicity assays or 1 × 10^6^ cells/mL in 6-well plates for a cell viability assay, protein isolation, and immunofluorescence. All hepatocytes were maintained in a 37°C incubator with 5% CO_2_. After 24-h culture, hepatocytes were treated with different concentrations of MCT (100, 200, 300, and 400 μM) for different times (6, 12, 24, 36, and 48 h) or pretreated with 0.5 mM of 4-PBA for 4 h or CHOP siRNA/siNC for 24 h before exposure to MCT.

### Cell Viability Assay

Cell Counting Kit-8 (Dojindo, Catalog No.: CK04, Japan) was performed to quantify cell viability according to the manufacturer’s instructions. Briefly, the hepatocytes were cultured in 96-well plates with appropriate treatment. After treating, the cells were incubated in the DMEM media with 10% CCK-8 reagent at 37°C for another 4 h. Absorbance was measured at 490 nm with a microplate spectrophotometer (Epoch Microplate Spectrophotometer, BioTek, United States).

### Apoptosis Detection by Annexin V/PI Double Staining

Cell apoptosis rate was measured with the Annexin V-FITC Apoptosis Detection Kit (Dojindo Catalog No.: AD10, Japan) according to the manufacturer’s instructions. Briefly, the hepatocytes were harvested and washed twice with cold PBS. Cells were resuspended in the binding buffer, and co-incubated with 5 μL of Annexin V-FITC and 5 μL of PI for 15 min at room temperature in the dark. The cells were analyzed by a BD FACSAria™ III flow cytometer (BD, United States) within 1 h, and the data were analyzed by Treestar Flowjo software (United States).

### Immunofluorescence Staining of GRP78 and CHOP Proteins

The treated hepatocytes were rinsed three times in PBS and fixed with 4% paraformaldehyde for 30 min. Then, they were mixed with 5% goat serum (Catalog No.:C0265, Beyotime Institute of Biotechnology, China), 0.1% Triton X-100, and GRP78 (1:100, Catalog No.: CY5166, Abways Technology, China) or CHOP (1:100, Catalog No.:BM4962, Boster Technology, China) was used to block cells and hatch the primary antibodies overnight at 4°C. The next day, cells were incubated with Alexa Fluor 488-conjugated donkey anti-rabbit secondary antibody (1:500, Catalog No.: ab150073, Abcam, United Kingdom) and DAPI (2 μg/ml, Catalog No.: D9542, Sigma, United States) at room temperature in the dark for 2 h. After rinsing with PBS five times, the hepatocytes were observed using laser confocal microscope (Carl Zeiss GmbH, Germany).

### Small Interfering RNA Transfection

Hepatocytes were seeded into 6-well plates or 96-well plates in Dulbecco's Modified Eagle Medium with 10% FBS. Twenty-four hours later, they were transfected with 100 nM of CHOP siRNA or 100 nM of negative control siRNA (siNC) using Advanced Transfection Reagent (Catalog No.: AD600050, Zeta life, United States) according to the manufacture’s protocol. The following CHOP siRNA sequences were used for transfection: 5′-CAG​UAU​CUU​GAG​UCU​AAU​ATT-3′ (sense) and 5′-UAU​UAG​ACU​CAA​GAU​ACU​GTT-3′ (antisense) (General Biosystems, China). After 24 h transfection, hepatocytes were exposed to 300 μM of MCT for another 36 h, followed by the cell viability assay, Annexin-V/PI staining, or western blotting.

### Western Blot Analysis

Hepatocytes were lysed in ice-cold radioimmunoprecipitation (RIPA) buffer containing a PMSF (R0010, Solarbio, China) and the lysates were centrifuged at 12,000 g for 10 min at 4°C to collect the supernatant. The protein concentrations were determined by the BCA method (PC0020; Solarbio, China). Each sample was separated by SDS-PAGE (10–15%) at 100 V for 1.5 h and then transferred to a polyvinylidene difluoride (PVDF) membrane (Catalog No. BSP0161, PALL, United States). The membranes were blocked with 5% non-fat milk in TBS-T for 2 h at room temperature and incubated with candidate primary antibodies in diluent overnight at 4°C. Glucose-regulated protein 78 (GRP78, 1:1,000, Catalog No.: CY5166) and phospho-IRE1 (1:1,000, Catalog No.: CY5166) were purchased from Abways Technology (China), IRE1 (1:1,000, Catalog No.: AI601) and phospho-eIF2α (1:1,000, Catalog No.: AF1237) were purchased from Beyotime Institute of Biotechnology (China), ATF 6 (1:1,000, Catalog No.: D262665) was purchased from Sangon Biotech (China), ATF4 (1:1,000, Catalog No.: OM108094) was purchased from OmnimAbs (United States), and CHOP (1:1,000, Catalog No.:BM4962) was purchased from Boster Technology (China). Caspase-3 (1:1,000, Catalog No.: ab32351), caspase-8 (1:1,000, Catalog No.: ab25901), and eIF2α (1:1,000, Catalog No.: ab115822) were purchased from Abcam (United States). *β*-actin (1:5,000, Catalog No.: 4,970) was obtained from Cell Signaling Technology (United States) to be used as the loading control antibody. The membranes were probed with goat anti-rabbit IgG-HRP secondary antibody (1:5,000, Beyotime Institute of Biotechnology, China).

### Statistical Analysis

All the results are presented in the way of the mean ± SD (vertical error bars) from triplicate experiments. The differences between groups were analyzed by one-way ANOVA implemented with GraphPad Prism version 7.0 software (GraphPad, San Diego, CA, United States). *P* < 0.05 indicated a statistical difference and *P* < 0.01 indicated a significant statistical difference.

## Result

### MCT Decreased Cell Viability in Primary Rat Hepatocytes

The toxic effects of MCT on primary rat hepatocytes were examined by exposure to 0–400 μM for 6–48 h. A CCK-8 assay was performed to assess the cell viability. MCT has no effect on the cell viability of primary rat hepatocytes when treated with different concentrations of MCT (100, 200, 300, and 400 μM) for 6, 12, and 24 h. However, it reduced the cell viability of primary rat hepatocytes after 36 h (MCT > 300 μM) and 48 h (MCT > 200 μM) (Figure 1B). These results indicated that MCT decreased the cell viability of primary rat hepatocytes based on a certain time and concentration.

### MCT Promoted Apoptosis in Primary Rat Hepatocytes

**FIGURE 2:**
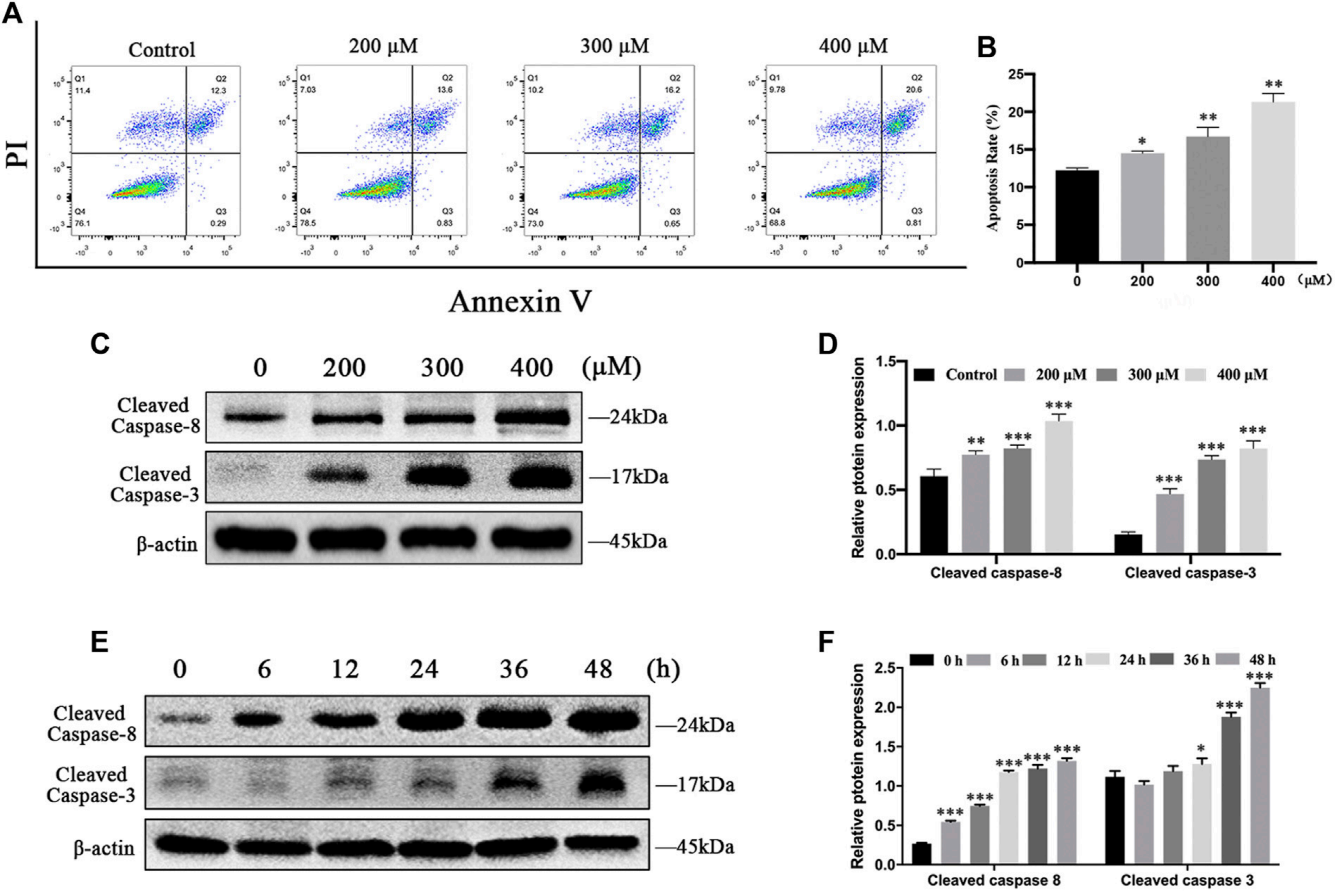
MCT induced apoptosis in primary rat hepatocytes via activation of caspase. **(A)** Representative images of hepatocytes that were treated with different doses of MCT (200, 300, and 400 μM) for 36 h followed by Annexin-V/PI staining. The percentages of apoptosis cells were measured by flow cytometry. The Q1 quadrant stands for cell death induced by mechanical damage or necrotic cells, the Q2 quadrant stands for late apoptosis cells, the Q3 quadrant stands for early apoptosis cells, and the Q4 quadrant stands for normal cells. The sum of cell apoptosis included early and late apoptosis cells. **(B)** The results of quantitative analyses of apoptosis rate. **(C)** Representative immunoblot against apoptosis-related proteins from hepatocytes treated with 300 μM of MCT for different times (6, 12, 24, 36, and 48 h). **(D)** Representative immunoblot against apoptosis-related proteins from hepatocytes treated with different doses of MCT (200, 300, and 400 μM) for 36 h *β*-actin served as a loading control. **(E)** Quantitative analysis of protein levels in C. **(F)** Quantitative analysis of protein levels in D. Data are presented as mean ± SD error of three independent experiments. *p < 0.05, **p < 0.01, ***p < 0.001 compared to control.

To further investigate whether MCT decreases cell survival by inducing apoptosis, we performed flow cytometry analysis in primary rat hepatocytes. The result showed that the rate of apoptosis was remarkably elevated by MCT (Figures 2A,B). In addition, to observe whether the apoptotic effect of MCT was activated by a cascade of caspases, the expression of cleaved caspase-8 and cleaved caspase-3 were detected by western blot. Consistently, MCT induced primary rat hepatocytes apoptosis in a dose- and time-dependent manner, as evidenced by increased expression of cleaved caspase-8 levels and cleaved caspase-3 (Figures 2C–F). Together, these results indicated that MCT triggers caspase-dependent apoptosis in primary rat hepatocytes.

### MCT Caused the Activation of the ER Stress Pathway in Primary Rat Hepatocytes

**FIGURE 3:**
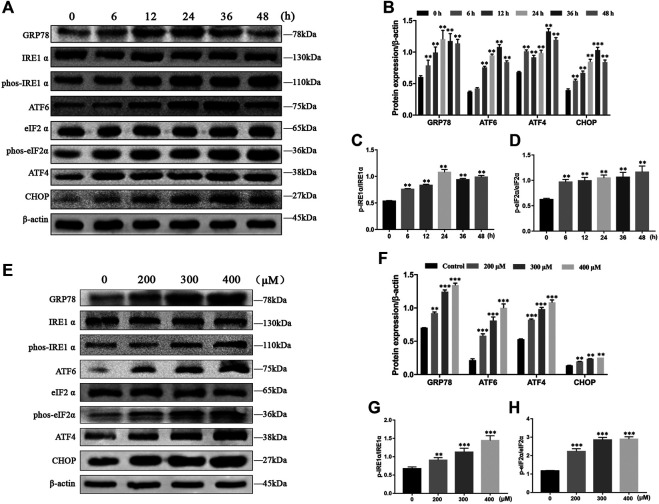
MCT induced ER stress in primary rat hepatocytes. **(A)** Representative immunoblot against ER stress-related proteins from hepatocytes treated with 300 μM of MCT for 6, 12, 24, 36, and 48 h. **(B–D)** Quantitative analysis of protein levels in A. **(E)** Representative immunoblot ER stress-related apoptosis-related proteins from hepatocytes treated with different doses of MCT (200, 300, and 400 μM) for 36 h. **(F–H)** Quantitative analysis of protein levels in E. *β*-actin served as a loading control. Data are presented as mean ± SD error of three independent experiments. *p < 0.05, **p < 0.01 compared to control.

To evaluate whether MCT activates ER stress in primary rat hepatocytes, we examined the expression of ER stress pathway-related proteins by western blot, including GRP78, p-IRE1α, ATF6, p-eIF2α, ATF4, and CHOP. The results showed that the expressions of GRP78, p-IRE1α, ATF6, ATF4, and CHOP at different times (0, 6, 12, 24, 36, and 48 h) increased first and then decreased with increasing time, and p-eIF2α levels was consistently increased after exposure to MCT (300 μM) (Figures 3A–D). In addition, we also detected the expressions of GRP78, p-IRE1α, ATF6, p-eIF2α, ATF4, and CHOP after exposure to 0, 200, 300, and 400 μM of MCT for 36 h, which were upregulated in a dose-dependent manner (Figures 3E–H). These results indicated that MCT induces ER stress in primary rat hepatocytes.

### Inhibition of ER Stress Ameliorated MCT-Induced Apoptosis in Primary Rat Hepatocytes

**FIGURE 4:**
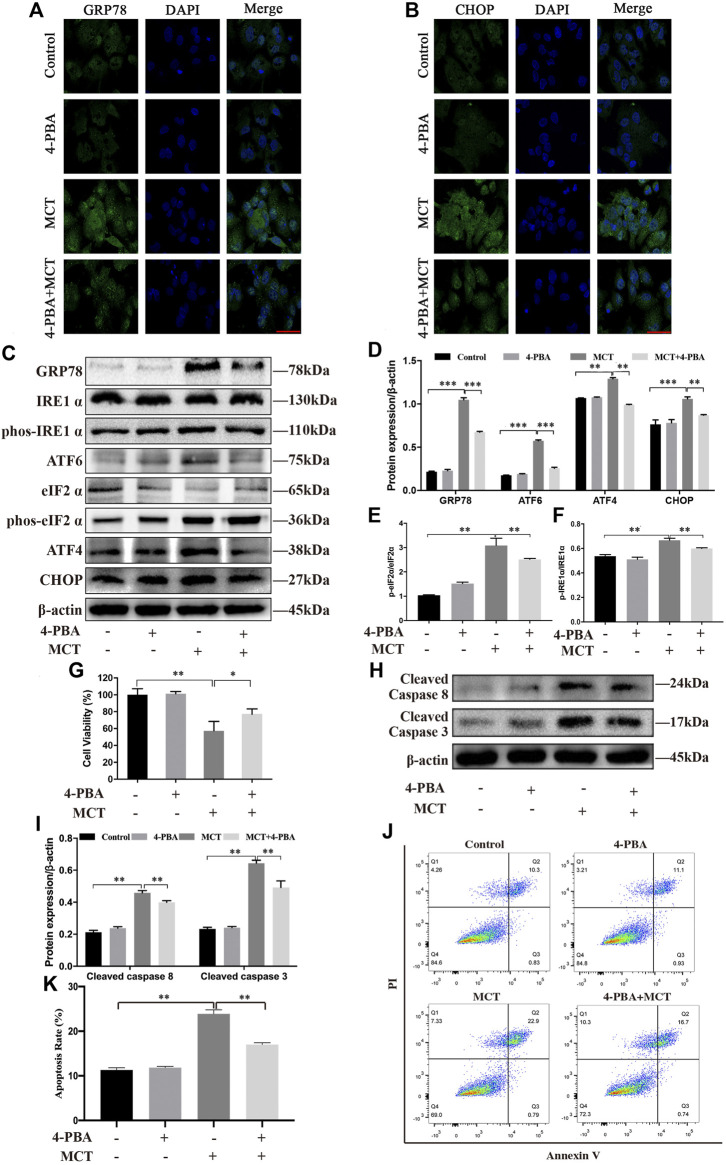
Inhibition of MCT-induced ER stress can partly protect primary rat hepatocytes from apoptosis. After pretreatment with 4-PBA (0.5 mM) for 4 h, the hepatocytes were treated with or without 300 μM of MCT for another 36 h. **(A)** Representative immunofluorescence photomicrographs showing the location of GRP78 in hepatocytes from different groups. **(B)** Representative immunofluorescence photomicrographs showing the location of CHOP in hepatocytes from different groups. Scale bar = 20 μM. **(C)** Detection of ER stress-related proteins, including GRP78, IRE1 α, p-IRE1 α, ATF6, eIF2 α, p-eIF2 α, ATF4, and CHOP by western blot. **(D–F)** Quantitative analysis of protein levels in C. **(G)** The hepatocytes viability was detected by CCK-8 assay. **(H)** Representative western blot of cleaved-caspase eight and cleaved-caspase three in hepatocytes. **(I)** Quantitative analysis of protein levels in G. **(J)** Representative apoptosis rate measured by Annexin-V/PI staining. The Q1 quadrant stands for cell death induced by mechanical damage or necrotic cells, the Q2 quadrant stands for late apoptosis cells, the Q3 quadrant stands for early apoptosis cells, and the Q4 quadrant stands for normal cells. The sum of cell apoptosis included early and late apoptosis cells. **(K)** The results of quantitative analyses of apoptosis rate. Data are presented as mean ± SD error of three independent experiments. *p < 0.05, **p < 0.01, ***p < 0.001 compared to control.

To explore whether ER stress mediated MCT-induced cell apoptosis, primary rat hepatocytes were treated with MCT in the presence or absence of 4-PBA (an ER stress inhibitor). We pretreated the hepatocytes with 0.5 mM of 4-PBA for 4 h and then exposed them to MCT (300 μM) for 36 h before subsequent experiments. As show in Figures 4A,B, 4-PBA significantly reduced the immunofluorescence staining of GRP78 and CHOP in primary rat hepatocytes. Consistently, western blot analysis also revealed that the expression of GRP78, p-IRE1α, ATF6, p-eIF2α, ATF4, and CHOP was markedly decreased in the 4-PBA + MCT-exposed primary rat hepatocytes (Figures 4C–F). In addition, the result showed that pretreatment with 4-PBA significantly promoted cell viability (Figure 4G) and attenuated MCT-induced apoptosis by inhibiting the expression of cleaved caspase-8 and cleaved caspase-3 (Figures 4H,I), observed by the CCK-8 assay and western blot analysis, respectively. Annexin V-FITC/PI double staining also showed that pretreatment with 4-PBA obviously decreased cell apoptosis rate induced by MCT (Figures 4J,K). These results suggested that inhibition of ER stress ameliorated MCT-induced apoptosis in primary rat hepatocytes.

### CHOP Is an Essential Part of the MCT-Induced Apoptosis in Primary Rat Hepatocytes

**FIGURE 5:**
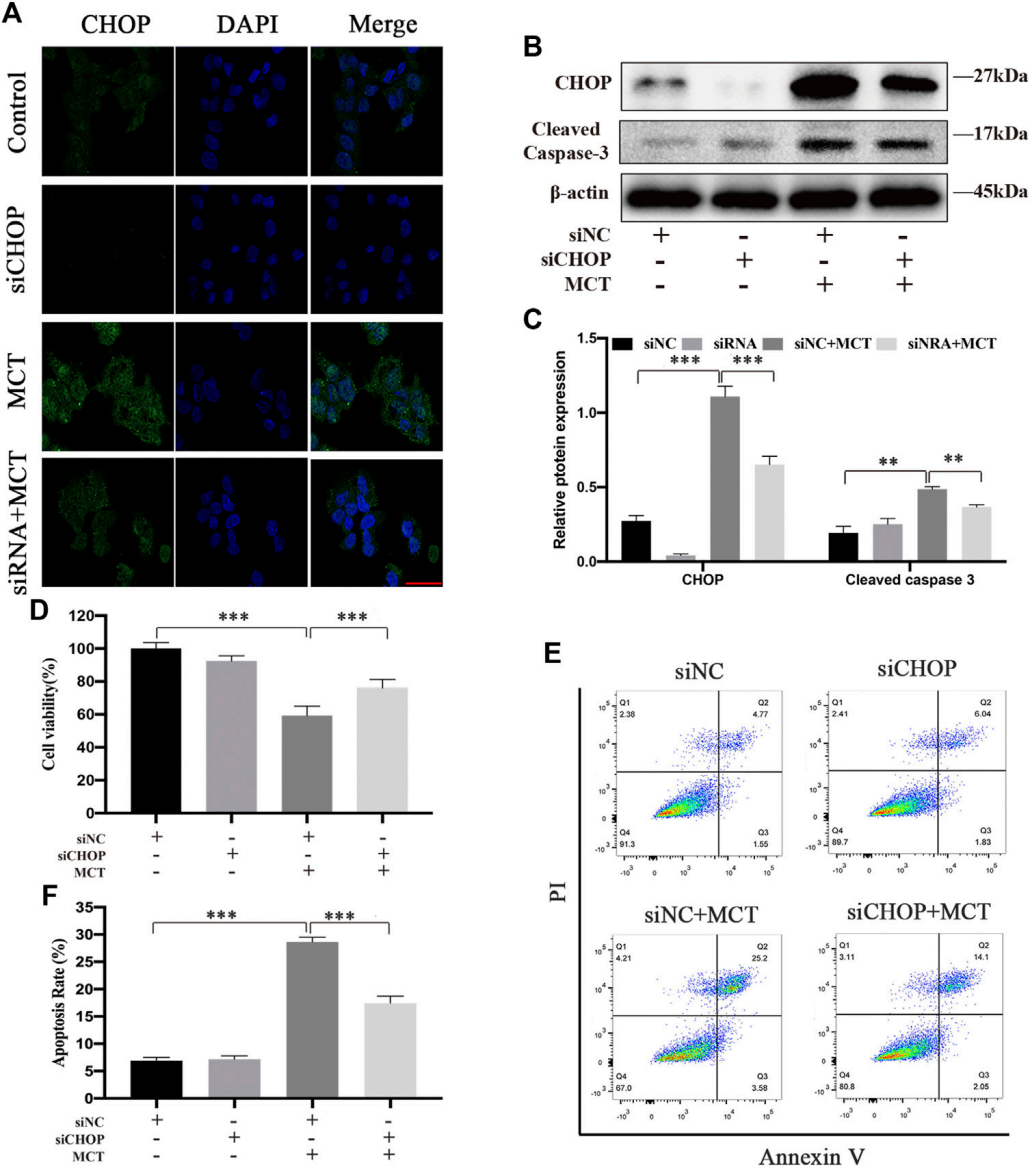
CHOP siRNA partially decreases MCT-induced apoptosis of primary rat hepatocytes. After pretreatment with CHOP siRNA (100 nM) or siNC (100 nM) for 24 h, the hepatocytes were treated with or without 300 μM of MCT for another 36 h. **(A)** Representative immunofluorescence photomicrographs showing the location of CHOP in hepatocytes from different groups. Scale bar = 20 μM. **(B)** Western blot was used to detect the expression of CHOP and cleaved caspase-3. **(C)** Quantitative analysis of protein levels in A. **(D)** The apoptosis rate of primary rat hepatocytes was detected by Annexin-V/PI staining. The Q1 quadrant stands for cell death induced by mechanical damage or necrotic cells, the Q2 quadrant stands for late apoptosis cells, the Q3 quadrant stands for early apoptosis cells, and the Q4 quadrant stands for normal cells. The sum of cell apoptosis included early and late apoptosis cells **(E)** The percentages of apoptosis cells were measured by flow cytometry. **(F)** Hepatocytes viability was detected by CCK-8 assay. Data are presented as mean ± SD error of three independent experiments. *p < 0.05, **p < 0.01, ***p < 0.001 compared to control.

CHOP has been reported to have an important role in regulating cell apoptosis after ER stress (Hu et al., 2018). To investigate the role of CHOP in the MCT-induced apoptosis of primary rat hepatocytes, we pretreated hepatocytes with CHOP siRNA or siNC for 24 h followed by MCT treatment. The immunofluorescence staining and western blot showed respectively that CHOP was knocked down with its siRNA (Figures 5A–C). As show in Figures 5A,D CCK-8 assay was performed to show that knockdown of CHOP significantly promoted cell viability. Meanwhile, knockdown of CHOP significantly decreased the expression of apoptosis-related proteins such as cleaved caspase-3 (Figures 5B,C). Furthermore, the flow cytometry assay revealed that MCT-induced apoptosis was significantly attenuated in hepatocytes with downregulated CHOP (Figures 5E,F). Altogether, the data suggested that knockdown of CHOP attenuated apoptosis induced by MCT.

## Discussion

MCT is a major pyrrolizidine alkaloid in *Crotalaria* sp., and has well-documented hepatotoxicity both for animals and humans (Williams and Molyneux, 1987; Huxtable, 1989; Xiang et al., 2020). The typical symptoms caused by MCT include hepatic sinusoidal obstruction syndrome (SOS) (Zhang et al., 2017). However, the underlying mechanisms involved in MCT-induced hepatotoxicity are not fully understood.

Apoptosis and ER stress are interrelated cellular processes of programmed cell death (Iurlaro and Muñoz-Pinedo, 2016). Crosstalk between these two pathways may reveal how MCT impacts hepatocyte function in pathologic states. As a major pathological cellular process, MCT-induced apoptosis has been found in the liver (Nakamura et al., 2012). Meanwhile, our previous study suggested that MCT could induce ER stress in liver (Guo et al., 2020). However, the interplay between apoptosis and ER stress in MCT-induced pathological processes is unclear. Therefore, in this study, we explored the effect of MCT on hepatocytes and the role of CHOP in apoptosis and ER stress.

MCT needs to be catalyzed by cytochrome P450 (CYP450) to exert its toxic effect (Fu et al., 2004; Maioli et al., 2011). Since this step is considered to occur in the liver, it takes a certain time for MCT to enter the liver and metabolize before it has toxic effects. In this study, our result showed that MCT had no effect on the cell viability of primary rat hepatocytes when treated with different concentrations of MCT for 6, 12, and 24 h. Previous studies have shown that the EC_50_ concentration of MCT was more than 300 μM after exposure to primary rat hepatocyte for 48 h (Gao et al., 2020). In this study, MCT decreased the cell viability obviously after 36 h (MCT＞300 μM), but also did not reach EC_50_ (Figure 1B). However, the CCK-8 assay performed for the EC_50_ concentration of MCT exposure to primary rat hepatocyte was 298.7 ± 2.4 μM for 48 h. This may be related to the fact that the primary rat hepatocytes were isolated from different species of rats, which may affect the activity of P450 enzymes.

Apoptosis is a form of programmed cell death that results in the orderly and efficient removal of damaged cells in response to many natural products. Sustained apoptosis causes cell death and ultimately leads to cell dysfunction (D’arcy, 2019). Previous studies have shown that some PAs can induce apoptosis in primary mouse hepatocytes (Yang et al., 2017b) or cell lines, including human live L-02 cells (Ji et al., 2008), human hepatoma cells HepG2 (Ebmeyer et al., 2019), and Huh-7 (Liu et al., 2017). In this study, we tested the toxic effects of MCT with different concentrations and time on primary rat hepatocytes. MCT was found to dramatically increase the apoptosis rate of primary rat hepatocytes in a dose-dependent manner (Figures 2A,B). We also found that MCT caused apoptosis by initiating the activation of cleaved caspase-8 and cleaved caspase-3 in a dose- and time-dependent manner (Figures 2C–F). These results suggested that MCT induced cytotoxicity by its involvement in the apoptosis pathway in primary rat hepatocytes.

ER stress is one of the vital mechanisms involved in response to liver disease (Malhi and Kaufman, 2011). Mild ERs could help cells to adapt to stress by modifying protein folding, and/or promoting ER-associated degradation (ERAD) pathways to remove misfolded proteins. However, sustained ER stress disorders intracellular homeostasis and activated apoptosis programs (Wang et al., 2019). Recently, several drugs have been proven to have cytotoxicity effects through ER stress-induced apoptosis (Guo et al., 2017; Wang et al., 2019; Wang and Tang, 2020). When ER homeostasis was disturbed by exogenously applied chemicals, three sensors (PERK, IRE1, and ATF6) were activated due to division from GRP78, which binds to the accumulation of unfolded proteins. IRE1α dimerized and transautophosphorylated leading to activation of the XBP1 and JNK pathway, which activated apoptosis. PERK phosphorylated eukaryotic initiation factor 2 alpha (eIF2α), which increased the translation of transcription factor ATF4, the upstream factor of CHOP. Activated ATF6 also transactivated the CHOP gene (Wu et al., 2016). It is well known that CHOP is associated with apoptosis (Hu et al., 2018). In our research, the expression of ER stress marker proteins GRP78, p-IRE1α, ATF6, ATF4, and CHOP increased first and then decreased with increasing time, and p-eIF2α levels was consistently increased (Figures 3A–D). Similarly, we also examined primary rat hepatocytes with increasing MCT concentrations, the levels of GRP78, p-IRE1α, ATF6, p-eIF2α, ATF4, and CHOP were significantly increased (Figures 3E–H). Taken together, these results suggested that MCT promotes ER stress in primary rat hepatocytes.

In order to clarify the relationship between apoptosis and ER stress, the role of ER stress in MCT-treated cell survival was further investigated. 4-PBA, a common chemical chaperone, acts as an inhibitor of ER stress through alleviating the production of misfolded proteins in the ER (Kolb et al., 2015). In this study, 4-PBA blocked ER stress confirmed by the decreased expression of GRP78, p-IRE 1α, ATF6, p-eIF2α, ATF4, and CHOP (Figures 4A–F). In order to investigate the interplay between MCT-induced ER stress and apoptosis, we detected the cell viability, the expression of apoptosis-related proteins, and apoptosis rate after pre-treating 4-PBA before exposure to MCT. Our result showed that inhibiting ER stress by 4-PBA obviously promoted cell viability (Figure 4G). Meanwhile, 4-PBA attenuated the expression of cleaved caspase-8 and cleaved caspase-3 (Figures 4H,I) and the apoptosis rate (Figures 4J,K). Therefore, these results suggested that ER stress was involved in MCT-induced apoptosis in primary rat hepatocytes.

CHOP is the most well characterized pro-apoptotic pathway that activates from the stressed ER (Hu et al., 2018). Though the exact mechanisms that mediate ER stress-induced apoptosis have not yet been elucidated, the three stress sensors have all been involved in apoptosis via activating CHOP (Wu et al., 2016). As reported, CHOP deficiency alleviated the damage of acute liver failure (Rao et al., 2015). In this study, we provided solid evidence that CHOP expression was knocked down by CHOP siRNA (Figures 5A–C). We also detected the cell viability, the expression of apoptosis-related proteins, and apoptosis rate of cells pretreated with CHOP siRNA or siNC for 24 h followed by MCT treatment. Our results showed that the expression of cleaved caspase-3 was blocked by knockdown of CHOP (Figures 5B,C). Meanwhile, CHOP siRNA significantly enhanced hepatocytes viability (Figure 5D) and decreased the apoptosis rate (Figures 5E,F). These data suggested that CHOP is a key factor in MCT-induced ER stress in primary rat hepatocytes.

## Conclusion

**FIGURE 6:**
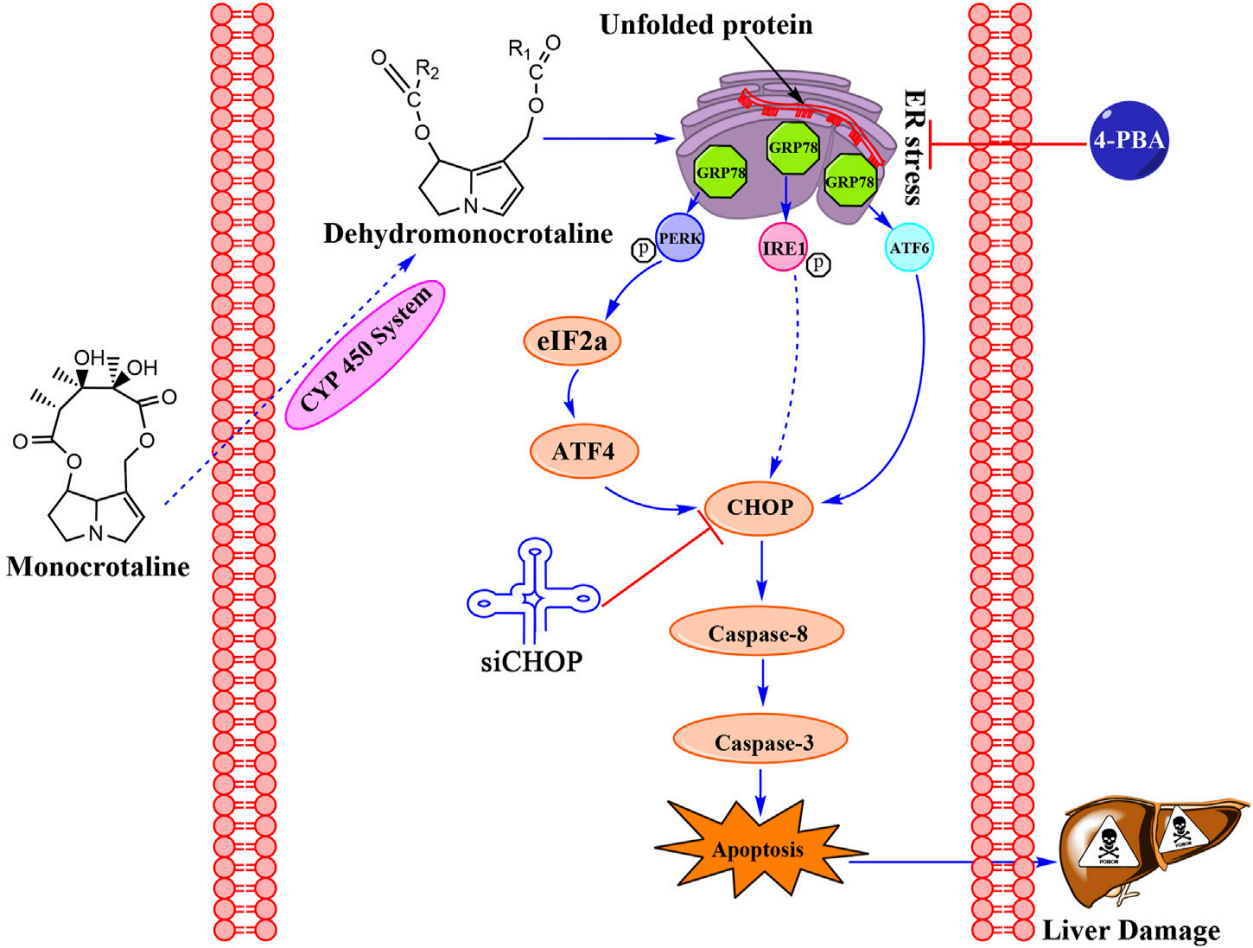
The signaling pathway involved in MCT-induced apoptosis in primary rat hepatocytes.

MCT can induce cytotoxicity via the ER stress pathway in primary rat hepatocytes. Moreover, the evidence further indicated that CHOP played a vital role in MCT-induced apoptosis mediated by ER stress (Figure 6). We believe that this work may provide another mechanistic insight for understanding the hepatotoxicity of MCT. 

## Data Availability

The datasets presented in this study can be found in online repositories. The names of the repository/repositories and accession number(s) can be found below: https://www.ncbi.nlm.nih.gov/gene/29467.
